# Supplementary material for: Comparative multiomics analysis of cell physiological state after culture in a basket bioreactor
Source: Sci Rep. 2022 Nov 23;12:20161. doi: 10.1038/s41598-022-24687-4 (PMC9686226; doi:10.1038/s41598-022-24687-4)
Supplement: Supplementary file 1 — Supplementary Information 1. [file 41598_2022_24687_MOESM1_ESM.zip › raw data/Transcriptomic raw data/6.Enrichment/2.KEGG/TE1vsTC1/UP/plot/TE1vsTC1.html]

### TE1vsTC1 KEGG通路富集列表

---

| KEGGID | Description | GeneRatio | BgRatio | pvalue | padj | geneID | geneName | keggID | Count |
| --- | --- | --- | --- | --- | --- | --- | --- | --- | --- |
| csab04630 | JAK-STAT signaling pathway | 7/87 | 111/5900 | 0.0011684 | 0.1728850 | 103226390/103235305/103246489/103236305/103237430/103247197/103238870 | IL6/IL11/CCND1/IL15/MYC/MCL1/SOCS2 | csab:103226390/csab:103235305/csab:103246489/csab:103236305/csab:103237430/csab:103247197/csab:103238870 | 7 |
| csab00900 | Terpenoid backbone biosynthesis | 3/87 | 18/5900 | 0.0021533 | 0.1728850 | 103240824/103215216/103223243 | ACAT2/HMGCS1/HMGCR | csab:103240824/csab:103215216/csab:103223243 | 3 |
| csab00100 | Steroid biosynthesis | 3/87 | 19/5900 | 0.0025300 | 0.1728850 | 103237411/103226567/103215320 | SQLE/CYP51A1/FDFT1 | csab:103237411/csab:103226567/csab:103215320 | 3 |
| csab00970 | Aminoacyl-tRNA biosynthesis | 4/87 | 43/5900 | 0.0035120 | 0.1799923 | 103226274/103219751/103229694/103238568 | GARS1/IARS1/WARS1/MARS1 | csab:103226274/csab:103219751/csab:103229694/csab:103238568 | 4 |
| csab04012 | ErbB signaling pathway | 5/87 | 79/5900 | 0.0059488 | 0.2258270 | 103235805/103244654/103235804/103237430/103228406 | AREG/HBEGF/EREG/MYC/CBLB | csab:103235805/csab:103244654/csab:103235804/csab:103237430/csab:103228406 | 5 |
| csab05210 | Colorectal cancer | 5/87 | 81/5900 | 0.0066096 | 0.2258270 | 103235805/103224652/103246489/103235804/103237430 | AREG/GADD45A/CCND1/EREG/MYC | csab:103235805/csab:103224652/csab:103246489/csab:103235804/csab:103237430 | 5 |
| csab04151 | PI3K-Akt signaling pathway | 10/87 | 280/5900 | 0.0077598 | 0.2272510 | 103226390/103238335/103243277/103235805/103246489/103235804/103225530/103237430/103247197/103214795 | IL6/NR4A1/ITGB3/AREG/CCND1/EREG/EPHA2/MYC/MCL1/COL4A1 | csab:103226390/csab:103238335/csab:103243277/csab:103235805/csab:103246489/csab:103235804/csab:103225530/csab:103237430/csab:103247197/csab:103214795 | 10 |
| csab05222 | Small cell lung cancer | 5/87 | 87/5900 | 0.0088992 | 0.2280432 | 103224652/103246489/103237430/103214795/103242634 | GADD45A/CCND1/MYC/COL4A1/TRAF4 | csab:103224652/csab:103246489/csab:103237430/csab:103214795/csab:103242634 | 5 |
| csab05216 | Thyroid cancer | 3/87 | 32/5900 | 0.0112839 | 0.2570211 | 103224652/103246489/103237430 | GADD45A/CCND1/MYC | csab:103224652/csab:103246489/csab:103237430 | 3 |
| csab05219 | Bladder cancer | 3/87 | 38/5900 | 0.0180166 | 0.3395277 | 103244654/103246489/103237430 | HBEGF/CCND1/MYC | csab:103244654/csab:103246489/csab:103237430 | 3 |
| csab05206 | MicroRNAs in cancer | 6/87 | 145/5900 | 0.0196508 | 0.3395277 | 103214617/103243277/103246489/103237430/103247197/103214788 | SPRY2/ITGB3/CCND1/MYC/MCL1/IRS2 | csab:103214617/csab:103243277/csab:103246489/csab:103237430/csab:103247197/csab:103214788 | 6 |
| csab00450 | Selenocompound metabolism | 2/87 | 15/5900 | 0.0199265 | 0.3395277 | 103224624/103238568 | CTH/MARS1 | csab:103224624/csab:103238568 | 2 |
| csab05410 | Hypertrophic cardiomyopathy | 4/87 | 74/5900 | 0.0232150 | 0.3395277 | 103226390/103243277/103222156/103217090 | IL6/ITGB3/EDN1/CACNB4 | csab:103226390/csab:103243277/csab:103222156/csab:103217090 | 4 |
| csab00270 | Cysteine and methionine metabolism | 3/87 | 42/5900 | 0.0235096 | 0.3395277 | 103218755/103224624/103224094 | BCAT1/CTH/PHGDH | csab:103218755/csab:103224624/csab:103224094 | 3 |
| csab04350 | TGF-beta signaling pathway | 4/87 | 77/5900 | 0.0264163 | 0.3395277 | 103246725/103237430/103237062/103245923 | BMP2/MYC/E2F5/GREM1 | csab:103246725/csab:103237430/csab:103237062/csab:103245923 | 4 |
| csab00600 | Sphingolipid metabolism | 3/87 | 44/5900 | 0.0265599 | 0.3395277 | 103236153/103230017/103219011 | UGT8/DEGS1/UGCG | csab:103236153/csab:103230017/csab:103219011 | 3 |
| csab04010 | MAPK signaling pathway | 8/87 | 247/5900 | 0.0284586 | 0.3395277 | 103238335/103216504/103235805/103224652/103235804/103225530/103237430/103217090 | NR4A1/DUSP5/AREG/GADD45A/EREG/EPHA2/MYC/CACNB4 | csab:103238335/csab:103216504/csab:103235805/csab:103224652/csab:103235804/csab:103225530/csab:103237430/csab:103217090 | 8 |
| csab00280 | Valine, leucine and isoleucine degradation | 3/87 | 46/5900 | 0.0298122 | 0.3395277 | 103218755/103240824/103215216 | BCAT1/ACAT2/HMGCS1 | csab:103218755/csab:103240824/csab:103215216 | 3 |
| csab04625 | C-type lectin receptor signaling pathway | 4/87 | 83/5900 | 0.0335819 | 0.3623313 | 103226390/103215465/103216128/103228406 | IL6/EGR3/EGR2/CBLB | csab:103226390/csab:103215465/csab:103216128/csab:103228406 | 4 |
| csab00650 | Butanoate metabolism | 2/87 | 21/5900 | 0.0376477 | 0.3858889 | 103240824/103215216 | ACAT2/HMGCS1 | csab:103240824/csab:103215216 | 2 |
| csab05213 | Endometrial cancer | 3/87 | 55/5900 | 0.0469117 | 0.3999199 | 103224652/103246489/103237430 | GADD45A/CCND1/MYC | csab:103224652/csab:103246489/csab:103237430 | 3 |
| csab04218 | Cellular senescence | 5/87 | 134/5900 | 0.0471569 | 0.3999199 | 103226390/103224652/103246489/103237430/103237062 | IL6/GADD45A/CCND1/MYC/E2F5 | csab:103226390/csab:103224652/csab:103246489/csab:103237430/csab:103237062 | 5 |
| csab00601 | Glycosphingolipid biosynthesis - lacto and neolacto series | 2/87 | 24/5900 | 0.0480985 | 0.3999199 | 103234996/103217238 | FUT1/B3GALT1 | csab:103234996/csab:103217238 | 2 |
| csab04640 | Hematopoietic cell lineage | 3/87 | 56/5900 | 0.0490552 | 0.3999199 | 103226390/103235305/103243277 | IL6/IL11/ITGB3 | csab:103226390/csab:103235305/csab:103243277 | 3 |
| csab04933 | AGE-RAGE signaling pathway in diabetic complications | 4/87 | 95/5900 | 0.0510192 | 0.3999199 | 103226390/103222156/103246489/103214795 | IL6/EDN1/CCND1/COL4A1 | csab:103226390/csab:103222156/csab:103246489/csab:103214795 | 4 |
| csab01230 | Biosynthesis of amino acids | 3/87 | 57/5900 | 0.0512461 | 0.3999199 | 103218755/103224624/103224094 | BCAT1/CTH/PHGDH | csab:103218755/csab:103224624/csab:103224094 | 3 |
| csab04668 | TNF signaling pathway | 4/87 | 98/5900 | 0.0560273 | 0.3999199 | 103226390/103222156/103236305/103240296 | IL6/EDN1/IL15/TNFAIP3 | csab:103226390/csab:103222156/csab:103236305/csab:103240296 | 4 |
| csab04931 | Insulin resistance | 4/87 | 99/5900 | 0.0577539 | 0.3999199 | 103226390/103247816/103214788/103220112 | IL6/TRIB3/IRS2/GFPT1 | csab:103226390/csab:103247816/csab:103214788/csab:103220112 | 4 |
| csab04390 | Hippo signaling pathway | 5/87 | 142/5900 | 0.0578420 | 0.3999199 | 103246725/103248148/103235805/103246489/103237430 | BMP2/DLG2/AREG/CCND1/MYC | csab:103246725/csab:103248148/csab:103235805/csab:103246489/csab:103237430 | 5 |
| csab04115 | p53 signaling pathway | 3/87 | 61/5900 | 0.0604757 | 0.3999199 | 103225266/103224652/103246489 | SESN2/GADD45A/CCND1 | csab:103225266/csab:103224652/csab:103246489 | 3 |
| csab05323 | Rheumatoid arthritis | 3/87 | 61/5900 | 0.0604757 | 0.3999199 | 103226390/103235305/103236305 | IL6/IL11/IL15 | csab:103226390/csab:103235305/csab:103236305 | 3 |
| csab05207 | Chemical carcinogenesis - receptor activation | 5/87 | 146/5900 | 0.0636679 | 0.4078726 | 103219053/103246489/103214580/103237430/103218702 | KLF4/CCND1/KLF5/MYC/MGST1 | csab:103219053/csab:103246489/csab:103214580/csab:103237430/csab:103218702 | 5 |
| csab05202 | Transcriptional misregulation in cancer | 5/87 | 148/5900 | 0.0667015 | 0.4142693 | 103226390/103219133/103224652/103228427/103237430 | IL6/NR4A3/GADD45A/NFKBIZ/MYC | csab:103226390/csab:103219133/csab:103224652/csab:103228427/csab:103237430 | 5 |
| csab04919 | Thyroid hormone signaling pathway | 4/87 | 105/5900 | 0.0687081 | 0.4142693 | 103243277/103246489/103237335/103237430 | ITGB3/CCND1/MED30/MYC | csab:103243277/csab:103246489/csab:103237335/csab:103237430 | 4 |
| csab04672 | Intestinal immune network for IgA production | 2/87 | 31/5900 | 0.0758845 | 0.4275575 | 103226390/103236305 | IL6/IL15 | csab:103226390/csab:103236305 | 2 |
| csab05162 | Measles | 4/87 | 110/5900 | 0.0786020 | 0.4275575 | 103226390/103246489/103240296/103228406 | IL6/CCND1/TNFAIP3/CBLB | csab:103226390/csab:103246489/csab:103240296/csab:103228406 | 4 |
| csab04110 | Cell cycle | 4/87 | 111/5900 | 0.0806626 | 0.4275575 | 103224652/103246489/103237430/103237062 | GADD45A/CCND1/MYC/E2F5 | csab:103224652/csab:103246489/csab:103237430/csab:103237062 | 4 |
| csab04657 | IL-17 signaling pathway | 3/87 | 69/5900 | 0.0810711 | 0.4275575 | 103226390/103240296/103242634 | IL6/TNFAIP3/TRAF4 | csab:103226390/csab:103240296/csab:103242634 | 3 |
| csab05169 | Epstein-Barr virus infection | 5/87 | 157/5900 | 0.0813402 | 0.4275575 | 103226390/103224652/103246489/103237430/103240296 | IL6/GADD45A/CCND1/MYC/TNFAIP3 | csab:103226390/csab:103224652/csab:103246489/csab:103237430/csab:103240296 | 5 |
| csab04925 | Aldosterone synthesis and secretion | 3/87 | 70/5900 | 0.0838354 | 0.4296567 | 103238335/103234364/103238835 | NR4A1/LDLR/ATP2B1 | csab:103238335/csab:103234364/csab:103238835 | 3 |
| csab05220 | Chronic myeloid leukemia | 3/87 | 71/5900 | 0.0866401 | 0.4332006 | 103224652/103246489/103237430 | GADD45A/CCND1/MYC | csab:103224652/csab:103246489/csab:103237430 | 3 |
| csab04068 | FoxO signaling pathway | 4/87 | 117/5900 | 0.0935839 | 0.4470503 | 103226390/103224652/103246489/103214788 | IL6/GADD45A/CCND1/IRS2 | csab:103226390/csab:103224652/csab:103246489/csab:103214788 | 4 |
| csab05200 | Pathways in cancer | 10/87 | 428/5900 | 0.0973211 | 0.4470503 | 103226390/103246725/103224652/103222156/103246489/103236305/103237430/103214795/103218702/103242634 | IL6/BMP2/GADD45A/EDN1/CCND1/IL15/MYC/COL4A1/MGST1/TRAF4 | csab:103226390/csab:103246725/csab:103224652/csab:103222156/csab:103246489/csab:103236305/csab:103237430/csab:103214795/csab:103218702/csab:103242634 | 10 |
| csab04913 | Ovarian steroidogenesis | 2/87 | 36/5900 | 0.0981330 | 0.4470503 | 103234364/103237812 | LDLR/LOC103237812 | csab:103234364/csab:103237812 | 2 |
| csab04979 | Cholesterol metabolism | 2/87 | 36/5900 | 0.0981330 | 0.4470503 | 103234364/103229330 | LDLR/NPC2 | csab:103234364/csab:103229330 | 2 |
| csab00260 | Glycine, serine and threonine metabolism | 2/87 | 37/5900 | 0.1027823 | 0.4580516 | 103224624/103224094 | CTH/PHGDH | csab:103224624/csab:103224094 | 2 |
| csab04060 | Cytokine-cytokine receptor interaction | 5/87 | 170/5900 | 0.1052698 | 0.4591555 | 103226390/103235305/103246725/103236305/103221402 | IL6/IL11/BMP2/IL15/TNFRSF21 | csab:103226390/csab:103235305/csab:103246725/csab:103236305/csab:103221402 | 5 |
| csab05160 | Hepatitis C | 4/87 | 125/5900 | 0.1122429 | 0.4793706 | 103246489/103237430/103234364/103241901 | CCND1/MYC/LDLR/CLDN1 | csab:103246489/csab:103237430/csab:103234364/csab:103241901 | 4 |
| csab04930 | Type II diabetes mellitus | 2/87 | 41/5900 | 0.1219515 | 0.5102051 | 103238870/103214788 | SOCS2/IRS2 | csab:103238870/csab:103214788 | 2 |
| csab05166 | Human T-cell leukemia virus 1 infection | 5/87 | 183/5900 | 0.1323102 | 0.5424718 | 103226390/103246489/103236305/103237430/103216128 | IL6/CCND1/IL15/MYC/EGR2 | csab:103226390/csab:103246489/csab:103236305/csab:103237430/csab:103216128 | 5 |
| csab00480 | Glutathione metabolism | 2/87 | 44/5900 | 0.1368560 | 0.5501074 | 103220822/103218702 | ODC1/MGST1 | csab:103220822/csab:103218702 | 2 |
| csab05161 | Hepatitis B | 4/87 | 138/5900 | 0.1457574 | 0.5746204 | 103226390/103215465/103237430/103216128 | IL6/EGR3/MYC/EGR2 | csab:103226390/csab:103215465/csab:103237430/csab:103216128 | 4 |
| csab04310 | Wnt signaling pathway | 4/87 | 141/5900 | 0.1540014 | 0.5956658 | 103232583/103246489/103237430/103231576 | GPC4/CCND1/MYC/TBL1X | csab:103232583/csab:103246489/csab:103237430/csab:103231576 | 4 |
| csab04927 | Cortisol synthesis and secretion | 2/87 | 50/5900 | 0.1677432 | 0.6266501 | 103238335/103234364 | NR4A1/LDLR | csab:103238335/csab:103234364 | 2 |
| csab05225 | Hepatocellular carcinoma | 4/87 | 146/5900 | 0.1681256 | 0.6266501 | 103224652/103246489/103237430/103218702 | GADD45A/CCND1/MYC/MGST1 | csab:103224652/csab:103246489/csab:103237430/csab:103218702 | 4 |
| csab04340 | Hedgehog signaling pathway | 2/87 | 52/5900 | 0.1782948 | 0.6412358 | 103219690/103246489 | GAS1/CCND1 | csab:103219690/csab:103246489 | 2 |
| csab04976 | Bile secretion | 2/87 | 52/5900 | 0.1782948 | 0.6412358 | 103234364/103223243 | LDLR/HMGCR | csab:103234364/csab:103223243 | 2 |
| csab04970 | Salivary secretion | 2/87 | 53/5900 | 0.1836096 | 0.6489649 | 103238835/103244467 | ATP2B1/SLC12A2 | csab:103238835/csab:103244467 | 2 |
| csab01210 | 2-Oxocarboxylic acid metabolism | 1/87 | 14/5900 | 0.1879627 | 0.6512200 | 103218755 | BCAT1 | csab:103218755 | 1 |
| csab05217 | Basal cell carcinoma | 2/87 | 56/5900 | 0.1996874 | 0.6512200 | 103246725/103224652 | BMP2/GADD45A | csab:103246725/csab:103224652 | 2 |
| csab05221 | Acute myeloid leukemia | 2/87 | 57/5900 | 0.2050852 | 0.6512200 | 103246489/103237430 | CCND1/MYC | csab:103246489/csab:103237430 | 2 |
| csab04152 | AMPK signaling pathway | 3/87 | 107/5900 | 0.2090596 | 0.6512200 | 103246489/103223243/103214788 | CCND1/HMGCR/IRS2 | csab:103246489/csab:103223243/csab:103214788 | 3 |
| csab04210 | Apoptosis | 3/87 | 107/5900 | 0.2090596 | 0.6512200 | 103224652/103247197/103235922 | GADD45A/MCL1/PTPN13 | csab:103224652/csab:103247197/csab:103235922 | 3 |
| csab05218 | Melanoma | 2/87 | 58/5900 | 0.2104996 | 0.6512200 | 103224652/103246489 | GADD45A/CCND1 | csab:103224652/csab:103246489 | 2 |
| csab00603 | Glycosphingolipid biosynthesis - globo and isoglobo series | 1/87 | 16/5900 | 0.2117925 | 0.6512200 | 103234996 | FUT1 | csab:103234996 | 1 |
| csab04917 | Prolactin signaling pathway | 2/87 | 60/5900 | 0.2213711 | 0.6512200 | 103246489/103238870 | CCND1/SOCS2 | csab:103246489/csab:103238870 | 2 |
| csab05230 | Central carbon metabolism in cancer | 2/87 | 60/5900 | 0.2213711 | 0.6512200 | 103237430/103233435 | MYC/SLC7A5 | csab:103237430/csab:103233435 | 2 |
| csab00532 | Glycosaminoglycan biosynthesis - chondroitin sulfate / dermatan sulfate | 1/87 | 17/5900 | 0.2234469 | 0.6512200 | 103215414 | CSGALNACT1 | csab:103215414 | 1 |
| csab04910 | Insulin signaling pathway | 3/87 | 111/5900 | 0.2245226 | 0.6512200 | 103238870/103214788/103228406 | SOCS2/IRS2/CBLB | csab:103238870/csab:103214788/csab:103228406 | 3 |
| csab05418 | Fluid shear stress and atherosclerosis | 3/87 | 111/5900 | 0.2245226 | 0.6512200 | 103243277/103222156/103218702 | ITGB3/EDN1/MGST1 | csab:103243277/csab:103222156/csab:103218702 | 3 |
| csab05214 | Glioma | 2/87 | 62/5900 | 0.2322895 | 0.6512200 | 103224652/103246489 | GADD45A/CCND1 | csab:103224652/csab:103246489 | 2 |
| csab05332 | Graft-versus-host disease | 1/87 | 18/5900 | 0.2349308 | 0.6512200 | 103226390 | IL6 | csab:103226390 | 1 |
| csab04972 | Pancreatic secretion | 2/87 | 63/5900 | 0.2377624 | 0.6512200 | 103238835/103244467 | ATP2B1/SLC12A2 | csab:103238835/csab:103244467 | 2 |
| csab05223 | Non-small cell lung cancer | 2/87 | 63/5900 | 0.2377624 | 0.6512200 | 103224652/103246489 | GADD45A/CCND1 | csab:103224652/csab:103246489 | 2 |
| csab05205 | Proteoglycans in cancer | 4/87 | 169/5900 | 0.2382512 | 0.6512200 | 103243277/103244654/103246489/103237430 | ITGB3/HBEGF/CCND1/MYC | csab:103243277/csab:103244654/csab:103246489/csab:103237430 | 4 |
| csab05163 | Human cytomegalovirus infection | 4/87 | 173/5900 | 0.2511293 | 0.6741428 | 103226390/103243277/103246489/103237430 | IL6/ITGB3/CCND1/MYC | csab:103226390/csab:103243277/csab:103246489/csab:103237430 | 4 |
| csab00770 | Pantothenate and CoA biosynthesis | 1/87 | 20/5900 | 0.2573974 | 0.6741428 | 103218755 | BCAT1 | csab:103218755 | 1 |
| csab04727 | GABAergic synapse | 2/87 | 67/5900 | 0.2597136 | 0.6741428 | 103238195/103238196 | SLC38A1/SLC38A2 | csab:103238195/csab:103238196 | 2 |
| csab05412 | Arrhythmogenic right ventricular cardiomyopathy | 2/87 | 68/5900 | 0.2652095 | 0.6741428 | 103243277/103217090 | ITGB3/CACNB4 | csab:103243277/csab:103217090 | 2 |
| csab05143 | African trypanosomiasis | 1/87 | 21/5900 | 0.2683849 | 0.6741428 | 103226390 | IL6 | csab:103226390 | 1 |
| csab03018 | RNA degradation | 2/87 | 69/5900 | 0.2707062 | 0.6741428 | 103242916/103234733 | TOB1/EXOSC5 | csab:103242916/csab:103234733 | 2 |
| csab00790 | Folate biosynthesis | 1/87 | 22/5900 | 0.2792117 | 0.6741428 | 103237812 | LOC103237812 | csab:103237812 | 1 |
| csab05224 | Breast cancer | 3/87 | 125/5900 | 0.2801478 | 0.6741428 | 103224652/103246489/103237430 | GADD45A/CCND1/MYC | csab:103224652/csab:103246489/csab:103237430 | 3 |
| csab04510 | Focal adhesion | 4/87 | 182/5900 | 0.2806234 | 0.6741428 | 103243277/103246489/103214795/103224044 | ITGB3/CCND1/COL4A1/PIP5K1A | csab:103243277/csab:103246489/csab:103214795/csab:103224044 | 4 |
| csab04934 | Cushing syndrome | 3/87 | 127/5900 | 0.2882246 | 0.6741428 | 103238335/103246489/103234364 | NR4A1/CCND1/LDLR | csab:103238335/csab:103246489/csab:103234364 | 3 |
| csab05226 | Gastric cancer | 3/87 | 127/5900 | 0.2882246 | 0.6741428 | 103224652/103246489/103237430 | GADD45A/CCND1/MYC | csab:103224652/csab:103246489/csab:103237430 | 3 |
| csab00630 | Glyoxylate and dicarboxylate metabolism | 1/87 | 23/5900 | 0.2898800 | 0.6741428 | 103240824 | ACAT2 | csab:103240824 | 1 |
| csab04975 | Fat digestion and absorption | 1/87 | 23/5900 | 0.2898800 | 0.6741428 | 103240824 | ACAT2 | csab:103240824 | 1 |
| csab05212 | Pancreatic cancer | 2/87 | 73/5900 | 0.2926766 | 0.6741428 | 103224652/103246489 | GADD45A/CCND1 | csab:103224652/csab:103246489 | 2 |
| csab04974 | Protein digestion and absorption | 2/87 | 75/5900 | 0.3036366 | 0.6761195 | 103214795/103238196 | COL4A1/SLC38A2 | csab:103214795/csab:103238196 | 2 |
| csab05146 | Amoebiasis | 2/87 | 75/5900 | 0.3036366 | 0.6761195 | 103226390/103214795 | IL6/COL4A1 | csab:103226390/csab:103214795 | 2 |
| csab04211 | Longevity regulating pathway | 2/87 | 76/5900 | 0.3091066 | 0.6761195 | 103225266/103214788 | SESN2/IRS2 | csab:103225266/csab:103214788 | 2 |
| csab05414 | Dilated cardiomyopathy | 2/87 | 76/5900 | 0.3091066 | 0.6761195 | 103243277/103217090 | ITGB3/CACNB4 | csab:103243277/csab:103217090 | 2 |
| csab01523 | Antifolate resistance | 1/87 | 25/5900 | 0.3107507 | 0.6761195 | 103226390 | IL6 | csab:103226390 | 1 |
| csab04666 | Fc gamma R-mediated phagocytosis | 2/87 | 77/5900 | 0.3145686 | 0.6761195 | 103240591/103224044 | WASF1/PIP5K1A | csab:103240591/csab:103224044 | 2 |
| csab04530 | Tight junction | 3/87 | 134/5900 | 0.3166218 | 0.6761195 | 103248148/103246489/103241901 | DLG2/CCND1/CLDN1 | csab:103248148/csab:103246489/csab:103241901 | 3 |
| csab04064 | NF-kappa B signaling pathway | 2/87 | 79/5900 | 0.3254650 | 0.6878384 | 103224652/103240296 | GADD45A/TNFAIP3 | csab:103224652/csab:103240296 | 2 |
| csab04512 | ECM-receptor interaction | 2/87 | 81/5900 | 0.3363186 | 0.6988958 | 103243277/103214795 | ITGB3/COL4A1 | csab:103243277/csab:103214795 | 2 |
| csab00250 | Alanine, aspartate and glutamate metabolism | 1/87 | 28/5900 | 0.3409248 | 0.6988958 | 103220112 | GFPT1 | csab:103220112 | 1 |
| csab04710 | Circadian rhythm | 1/87 | 28/5900 | 0.3409248 | 0.6988958 | 103227939 | BHLHE40 | csab:103227939 | 1 |
| csab04066 | HIF-1 signaling pathway | 2/87 | 83/5900 | 0.3471225 | 0.7045556 | 103226390/103222156 | IL6/EDN1 | csab:103226390/csab:103222156 | 2 |
| csab05231 | Choline metabolism in cancer | 2/87 | 85/5900 | 0.3578705 | 0.7192494 | 103240591/103224044 | WASF1/PIP5K1A | csab:103240591/csab:103224044 | 2 |
| csab01522 | Endocrine resistance | 2/87 | 87/5900 | 0.3685565 | 0.7235703 | 103244654/103246489 | HBEGF/CCND1 | csab:103244654/csab:103246489 | 2 |
| csab04724 | Glutamatergic synapse | 2/87 | 87/5900 | 0.3685565 | 0.7235703 | 103238195/103238196 | SLC38A1/SLC38A2 | csab:103238195/csab:103238196 | 2 |
| csab05167 | Kaposi sarcoma-associated herpesvirus infection | 3/87 | 148/5900 | 0.3734835 | 0.7235703 | 103226390/103246489/103237430 | IL6/CCND1/MYC | csab:103226390/csab:103246489/csab:103237430 | 3 |
| csab01200 | Carbon metabolism | 2/87 | 89/5900 | 0.3791750 | 0.7235703 | 103224094/103240824 | PHGDH/ACAT2 | csab:103224094/csab:103240824 | 2 |
| csab05203 | Viral carcinogenesis | 3/87 | 155/5900 | 0.4016565 | 0.7235703 | 103215465/103246489/103216128 | EGR3/CCND1/EGR2 | csab:103215465/csab:103246489/csab:103216128 | 3 |
| csab00620 | Pyruvate metabolism | 1/87 | 35/5900 | 0.4063468 | 0.7235703 | 103240824 | ACAT2 | csab:103240824 | 1 |
| csab00830 | Retinol metabolism | 1/87 | 35/5900 | 0.4063468 | 0.7235703 | 103231285 | ALDH1A3 | csab:103231285 | 1 |
| csab01250 | Biosynthesis of nucleotide sugars | 1/87 | 35/5900 | 0.4063468 | 0.7235703 | 103220112 | GFPT1 | csab:103220112 | 1 |
| csab05144 | Malaria | 1/87 | 35/5900 | 0.4063468 | 0.7235703 | 103226390 | IL6 | csab:103226390 | 1 |
| csab00565 | Ether lipid metabolism | 1/87 | 36/5900 | 0.4151529 | 0.7235703 | 103236153 | UGT8 | csab:103236153 | 1 |
| csab00982 | Drug metabolism - cytochrome P450 | 1/87 | 36/5900 | 0.4151529 | 0.7235703 | 103218702 | MGST1 | csab:103218702 | 1 |
| csab04978 | Mineral absorption | 1/87 | 36/5900 | 0.4151529 | 0.7235703 | 103238835 | ATP2B1 | csab:103238835 | 1 |
| csab00071 | Fatty acid degradation | 1/87 | 37/5900 | 0.4238299 | 0.7235703 | 103240824 | ACAT2 | csab:103240824 | 1 |
| csab04270 | Vascular smooth muscle contraction | 2/87 | 99/5900 | 0.4310852 | 0.7235703 | 103222156/103239748 | EDN1/CALCB | csab:103222156/csab:103239748 | 2 |
| csab00330 | Arginine and proline metabolism | 1/87 | 38/5900 | 0.4323795 | 0.7235703 | 103220822 | ODC1 | csab:103220822 | 1 |
| csab00380 | Tryptophan metabolism | 1/87 | 38/5900 | 0.4323795 | 0.7235703 | 103240824 | ACAT2 | csab:103240824 | 1 |
| csab00514 | Other types of O-glycan biosynthesis | 1/87 | 38/5900 | 0.4323795 | 0.7235703 | 103227904 | GXYLT2 | csab:103227904 | 1 |
| csab04961 | Endocrine and other factor-regulated calcium reabsorption | 1/87 | 38/5900 | 0.4323795 | 0.7235703 | 103238835 | ATP2B1 | csab:103238835 | 1 |
| csab05204 | Chemical carcinogenesis - DNA adducts | 1/87 | 38/5900 | 0.4323795 | 0.7235703 | 103218702 | MGST1 | csab:103218702 | 1 |
| csab05171 | Coronavirus disease - COVID-19 | 3/87 | 163/5900 | 0.4334309 | 0.7235703 | 103226390/103244654/103221259 | IL6/HBEGF/RPL22L1 | csab:103226390/csab:103244654/csab:103221259 | 3 |
| csab00140 | Steroid hormone biosynthesis | 1/87 | 39/5900 | 0.4408038 | 0.7235703 | 103237812 | LOC103237812 | csab:103237812 | 1 |
| csab04623 | Cytosolic DNA-sensing pathway | 1/87 | 39/5900 | 0.4408038 | 0.7235703 | 103226390 | IL6 | csab:103226390 | 1 |
| csab04936 | Alcoholic liver disease | 2/87 | 101/5900 | 0.4412014 | 0.7235703 | 103226390/103246489 | IL6/CCND1 | csab:103226390/csab:103246489 | 2 |
| csab04935 | Growth hormone synthesis, secretion and action | 2/87 | 102/5900 | 0.4462234 | 0.7249324 | 103238870/103214788 | SOCS2/IRS2 | csab:103238870/csab:103214788 | 2 |
| csab05416 | Viral myocarditis | 1/87 | 40/5900 | 0.4491044 | 0.7249324 | 103246489 | CCND1 | csab:103246489 | 1 |
| csab04514 | Cell adhesion molecules | 2/87 | 106/5900 | 0.4660613 | 0.7463301 | 103220287/103241901 | NRXN1/CLDN1 | csab:103220287/csab:103241901 | 2 |
| csab00590 | Arachidonic acid metabolism | 1/87 | 43/5900 | 0.4732825 | 0.7463301 | 103237812 | LOC103237812 | csab:103237812 | 1 |
| csab05321 | Inflammatory bowel disease | 1/87 | 43/5900 | 0.4732825 | 0.7463301 | 103226390 | IL6 | csab:103226390 | 1 |
| csab00520 | Amino sugar and nucleotide sugar metabolism | 1/87 | 44/5900 | 0.4811064 | 0.7471728 | 103220112 | GFPT1 | csab:103220112 | 1 |
| csab05134 | Legionellosis | 1/87 | 44/5900 | 0.4811064 | 0.7471728 | 103226390 | IL6 | csab:103226390 | 1 |
| csab00980 | Metabolism of xenobiotics by cytochrome P450 | 1/87 | 45/5900 | 0.4888154 | 0.7482667 | 103218702 | MGST1 | csab:103218702 | 1 |
| csab04926 | Relaxin signaling pathway | 2/87 | 111/5900 | 0.4902715 | 0.7482667 | 103222156/103214795 | EDN1/COL4A1 | csab:103222156/csab:103214795 | 2 |
| csab05135 | Yersinia infection | 2/87 | 112/5900 | 0.4950324 | 0.7482667 | 103226390/103224044 | IL6/PIP5K1A | csab:103226390/csab:103224044 | 2 |
| csab00983 | Drug metabolism - other enzymes | 1/87 | 46/5900 | 0.4964111 | 0.7482667 | 103218702 | MGST1 | csab:103218702 | 1 |
| csab04810 | Regulation of actin cytoskeleton | 3/87 | 181/5900 | 0.5025113 | 0.7519329 | 103243277/103240591/103224044 | ITGB3/WASF1/PIP5K1A | csab:103243277/csab:103240591/csab:103224044 | 3 |
| csab04261 | Adrenergic signaling in cardiomyocytes | 2/87 | 115/5900 | 0.5091481 | 0.7519592 | 103217090/103238835 | CACNB4/ATP2B1 | csab:103217090/csab:103238835 | 2 |
| csab04923 | Regulation of lipolysis in adipocytes | 1/87 | 48/5900 | 0.5112694 | 0.7519592 | 103214788 | IRS2 | csab:103214788 | 1 |
| csab04550 | Signaling pathways regulating pluripotency of stem cells | 2/87 | 116/5900 | 0.5137970 | 0.7519592 | 103219053/103237430 | KLF4/MYC | csab:103219053/csab:103237430 | 2 |
| csab00240 | Pyrimidine metabolism | 1/87 | 49/5900 | 0.5185353 | 0.7519592 | 103225017 | CTPS1 | csab:103225017 | 1 |
| csab01212 | Fatty acid metabolism | 1/87 | 50/5900 | 0.5256943 | 0.7519592 | 103240824 | ACAT2 | csab:103240824 | 1 |
| csab04061 | Viral protein interaction with cytokine and cytokine receptor | 1/87 | 50/5900 | 0.5256943 | 0.7519592 | 103226390 | IL6 | csab:103226390 | 1 |
| csab04924 | Renin secretion | 1/87 | 51/5900 | 0.5327481 | 0.7519592 | 103222156 | EDN1 | csab:103222156 | 1 |
| csab01240 | Biosynthesis of cofactors | 2/87 | 121/5900 | 0.5366109 | 0.7519592 | 103218755/103225017 | BCAT1/CTPS1 | csab:103218755/csab:103225017 | 2 |
| csab04921 | Oxytocin signaling pathway | 2/87 | 122/5900 | 0.5410865 | 0.7519592 | 103246489/103217090 | CCND1/CACNB4 | csab:103246489/csab:103217090 | 2 |
| csab03320 | PPAR signaling pathway | 1/87 | 53/5900 | 0.5465460 | 0.7519592 | 103215216 | HMGCS1 | csab:103215216 | 1 |
| csab04213 | Longevity regulating pathway - multiple species | 1/87 | 53/5900 | 0.5465460 | 0.7519592 | 103214788 | IRS2 | csab:103214788 | 1 |
| csab05133 | Pertussis | 1/87 | 53/5900 | 0.5465460 | 0.7519592 | 103226390 | IL6 | csab:103226390 | 1 |
| csab04022 | cGMP-PKG signaling pathway | 2/87 | 127/5900 | 0.5630211 | 0.7676710 | 103238835/103214788 | ATP2B1/IRS2 | csab:103238835/csab:103214788 | 2 |
| csab05132 | Salmonella infection | 3/87 | 199/5900 | 0.5671864 | 0.7676710 | 103226390/103237430/103220717 | IL6/MYC/RHOB | csab:103226390/csab:103237430/csab:103220717 | 3 |
| csab00310 | Lysine degradation | 1/87 | 57/5900 | 0.5729447 | 0.7676710 | 103240824 | ACAT2 | csab:103240824 | 1 |
| csab04920 | Adipocytokine signaling pathway | 1/87 | 57/5900 | 0.5729447 | 0.7676710 | 103214788 | IRS2 | csab:103214788 | 1 |
| csab04621 | NOD-like receptor signaling pathway | 2/87 | 131/5900 | 0.5800317 | 0.7677108 | 103226390/103240296 | IL6/TNFAIP3 | csab:103226390/csab:103240296 | 2 |
| csab04150 | mTOR signaling pathway | 2/87 | 132/5900 | 0.5842092 | 0.7677108 | 103225266/103233435 | SESN2/SLC7A5 | csab:103225266/csab:103233435 | 2 |
| csab04932 | Non-alcoholic fatty liver disease | 2/87 | 132/5900 | 0.5842092 | 0.7677108 | 103226390/103214788 | IL6/IRS2 | csab:103226390/csab:103214788 | 2 |
| csab01524 | Platinum drug resistance | 1/87 | 61/5900 | 0.5978233 | 0.7747335 | 103218702 | MGST1 | csab:103218702 | 1 |
| csab04260 | Cardiac muscle contraction | 1/87 | 61/5900 | 0.5978233 | 0.7747335 | 103217090 | CACNB4 | csab:103217090 | 1 |
| csab05165 | Human papillomavirus infection | 4/87 | 281/5900 | 0.6008908 | 0.7747335 | 103243277/103248148/103246489/103214795 | ITGB3/DLG2/CCND1/COL4A1 | csab:103243277/csab:103248148/csab:103246489/csab:103214795 | 4 |
| csab04144 | Endocytosis | 3/87 | 214/5900 | 0.6170799 | 0.7906336 | 103234364/103224044/103228406 | LDLR/PIP5K1A/CBLB | csab:103234364/csab:103224044/csab:103228406 | 3 |
| csab05100 | Bacterial invasion of epithelial cells | 1/87 | 66/5900 | 0.6269151 | 0.7982459 | 103240591 | WASF1 | csab:103240591 | 1 |
| csab04520 | Adherens junction | 1/87 | 67/5900 | 0.6324787 | 0.8003589 | 103240591 | WASF1 | csab:103240591 | 1 |
| csab01521 | EGFR tyrosine kinase inhibitor resistance | 1/87 | 69/5900 | 0.6433612 | 0.8059386 | 103226390 | IL6 | csab:103226390 | 1 |
| csab00562 | Inositol phosphate metabolism | 1/87 | 70/5900 | 0.6486823 | 0.8059386 | 103224044 | PIP5K1A | csab:103224044 | 1 |
| csab01232 | Nucleotide metabolism | 1/87 | 70/5900 | 0.6486823 | 0.8059386 | 103225017 | CTPS1 | csab:103225017 | 1 |
| csab04912 | GnRH signaling pathway | 1/87 | 73/5900 | 0.6641793 | 0.8202215 | 103244654 | HBEGF | csab:103244654 | 1 |
| csab04620 | Toll-like receptor signaling pathway | 1/87 | 75/5900 | 0.6741333 | 0.8275288 | 103226390 | IL6 | csab:103226390 | 1 |
| csab04916 | Melanogenesis | 1/87 | 76/5900 | 0.6790003 | 0.8285420 | 103222156 | EDN1 | csab:103222156 | 1 |
| csab05417 | Lipid and atherosclerosis | 2/87 | 160/5900 | 0.6889855 | 0.8299495 | 103226390/103234364 | IL6/LDLR | csab:103226390/csab:103234364 | 2 |
| csab04024 | cAMP signaling pathway | 2/87 | 161/5900 | 0.6922993 | 0.8299495 | 103222156/103238835 | EDN1/ATP2B1 | csab:103222156/csab:103238835 | 2 |
| csab04360 | Axon guidance | 2/87 | 161/5900 | 0.6922993 | 0.8299495 | 103225530/103246295 | EPHA2/SLIT2 | csab:103225530/csab:103246295 | 2 |
| csab04660 | T cell receptor signaling pathway | 1/87 | 80/5900 | 0.6977601 | 0.8316327 | 103228406 | CBLB | csab:103228406 | 1 |
| csab04659 | Th17 cell differentiation | 1/87 | 81/5900 | 0.7022781 | 0.8321793 | 103226390 | IL6 | csab:103226390 | 1 |
| csab04070 | Phosphatidylinositol signaling system | 1/87 | 83/5900 | 0.7111148 | 0.8378077 | 103224044 | PIP5K1A | csab:103224044 | 1 |
| csab05215 | Prostate cancer | 1/87 | 85/5900 | 0.7196922 | 0.8418724 | 103246489 | CCND1 | csab:103246489 | 1 |
| csab05142 | Chagas disease | 1/87 | 86/5900 | 0.7238859 | 0.8418724 | 103226390 | IL6 | csab:103226390 | 1 |
| csab04015 | Rap1 signaling pathway | 2/87 | 172/5900 | 0.7268850 | 0.8418724 | 103243277/103225530 | ITGB3/EPHA2 | csab:103243277/csab:103225530 | 2 |
| csab04020 | Calcium signaling pathway | 2/87 | 176/5900 | 0.7386339 | 0.8475429 | 103215153/103238835 | GDNF/ATP2B1 | csab:103215153/csab:103238835 | 2 |
| csab05145 | Toxoplasmosis | 1/87 | 90/5900 | 0.7400497 | 0.8475429 | 103234364 | LDLR | csab:103234364 | 1 |
| csab04928 | Parathyroid hormone synthesis, secretion and action | 1/87 | 93/5900 | 0.7515553 | 0.8559379 | 103244654 | HBEGF | csab:103244654 | 1 |
| csab03010 | Ribosome | 1/87 | 95/5900 | 0.7589445 | 0.8595780 | 103221259 | RPL22L1 | csab:103221259 | 1 |
| csab05208 | Chemical carcinogenesis - reactive oxygen species | 2/87 | 188/5900 | 0.7713552 | 0.8598660 | 103218702/103237812 | MGST1/LOC103237812 | csab:103218702/csab:103237812 | 2 |
| csab04145 | Phagosome | 1/87 | 100/5900 | 0.7764806 | 0.8598660 | 103243277 | ITGB3 | csab:103243277 | 1 |
| csab04380 | Osteoclast differentiation | 1/87 | 101/5900 | 0.7798334 | 0.8598660 | 103243277 | ITGB3 | csab:103243277 | 1 |
| csab04670 | Leukocyte transendothelial migration | 1/87 | 101/5900 | 0.7798334 | 0.8598660 | 103241901 | CLDN1 | csab:103241901 | 1 |
| csab04071 | Sphingolipid signaling pathway | 1/87 | 102/5900 | 0.7831364 | 0.8598660 | 103230017 | DEGS1 | csab:103230017 | 1 |
| csab04915 | Estrogen signaling pathway | 1/87 | 103/5900 | 0.7863905 | 0.8598660 | 103244654 | HBEGF | csab:103244654 | 1 |
| csab04217 | Necroptosis | 1/87 | 105/5900 | 0.7927545 | 0.8598660 | 103240296 | TNFAIP3 | csab:103240296 | 1 |
| csab04611 | Platelet activation | 1/87 | 105/5900 | 0.7927545 | 0.8598660 | 103243277 | ITGB3 | csab:103243277 | 1 |
| csab04371 | Apelin signaling pathway | 1/87 | 113/5900 | 0.8163896 | 0.8795432 | 103246489 | CCND1 | csab:103246489 | 1 |
| csab04080 | Neuroactive ligand-receptor interaction | 2/87 | 210/5900 | 0.8222524 | 0.8795432 | 103222156/103239748 | EDN1/CALCB | csab:103222156/csab:103239748 | 2 |
| csab05164 | Influenza A | 1/87 | 116/5900 | 0.8245482 | 0.8795432 | 103226390 | IL6 | csab:103226390 | 1 |
| csab04613 | Neutrophil extracellular trap formation | 1/87 | 118/5900 | 0.8297870 | 0.8795432 | 103243277 | ITGB3 | csab:103243277 | 1 |
| csab04142 | Lysosome | 1/87 | 119/5900 | 0.8323482 | 0.8795432 | 103229330 | NPC2 | csab:103229330 | 1 |
| csab04140 | Autophagy - animal | 1/87 | 126/5900 | 0.8492402 | 0.8884632 | 103214788 | IRS2 | csab:103214788 | 1 |
| csab04072 | Phospholipase D signaling pathway | 1/87 | 128/5900 | 0.8537495 | 0.8884632 | 103224044 | PIP5K1A | csab:103224044 | 1 |
| csab05152 | Tuberculosis | 1/87 | 129/5900 | 0.8559539 | 0.8884632 | 103226390 | IL6 | csab:103226390 | 1 |
| csab04120 | Ubiquitin mediated proteolysis | 1/87 | 130/5900 | 0.8581255 | 0.8884632 | 103228406 | CBLB | csab:103228406 | 1 |
| csab04141 | Protein processing in endoplasmic reticulum | 1/87 | 147/5900 | 0.8904585 | 0.9173065 | 103233048 | HERPUD1 | csab:103233048 | 1 |
| csab05415 | Diabetic cardiomyopathy | 1/87 | 169/5900 | 0.9217078 | 0.9447505 | 103220112 | GFPT1 | csab:103220112 | 1 |
| csab04014 | Ras signaling pathway | 1/87 | 184/5900 | 0.9377779 | 0.9563645 | 103225530 | EPHA2 | csab:103225530 | 1 |
| csab05010 | Alzheimer disease | 2/87 | 302/5900 | 0.9423689 | 0.9563645 | 103226390/103214788 | IL6/IRS2 | csab:103226390/csab:103214788 | 2 |
| csab05020 | Prion disease | 1/87 | 211/5900 | 0.9589148 | 0.9683623 | 103226390 | IL6 | csab:103226390 | 1 |
| csab05168 | Herpes simplex virus 1 infection | 2/87 | 410/5900 | 0.9862360 | 0.9910705 | 103226390/103243277 | IL6/ITGB3 | csab:103226390/csab:103243277 | 2 |
| csab05022 | Pathways of neurodegeneration - multiple diseases | 1/87 | 373/5900 | 0.9967365 | 0.9967365 | 103226390 | IL6 | csab:103226390 | 1 |
